# The use of oral antibiotics and mechanical bowel preparation in elective colorectal resection for the reduction of surgical site infection

**DOI:** 10.1111/codi.14982

**Published:** 2020-02-14

**Authors:** S. E. Duff, C. L. F. Battersby, R. J. Davies, L. Hancock, J. Pipe, S. Buczacki, J. Kinross, A. G. Acheson, C. J. Walsh

**Affiliations:** ^1^ Wythenshawe Hospital Manchester University NHS Foundation Trust Manchester UK; ^2^ Wrexham Maelor Hospital Wrexham UK; ^3^ Cambridge Colorectal Unit Addenbrookes Hospital Cambridge University NHS Foundation Trust Cambridge UK; ^4^ Patient Liaison Group ACPGBI Sheffield UK; ^5^ Department of Surgery and Cancer St Mary's Hospital Imperial College London UK; ^6^ Gastrointestinal Surgery Nottingham Digestive Diseases Centre National Institute for Health Research (NIHR) Biomedical Research Centre Nottingham University Hospitals NHS Trust Queen’s Medical Centre University of Nottingham Nottingham UK; ^7^ Wirral University Teaching Hospitals NHS Foundation Trust Wirral UK

## Introduction

Surgical site infection (SSI) is a major cause of morbidity worldwide following elective colorectal resection, affecting up to 20% of patients 1, 2, 3. Reduction in SSI rates requires a multi‐faceted approach 4 and can be achieved with the use of SSI reduction bundles 5. Such bundles include prophylactic intravenous antibiotics 6 which represent an undisputed standard of care 4. They do not include the use of mechanical bowel preparation (MBP) alone which is not recommended in elective colonic resection to reduce SSI 7, 8, 9, although may offer an advantage in elective rectal resection 10.

**Figure 1:**
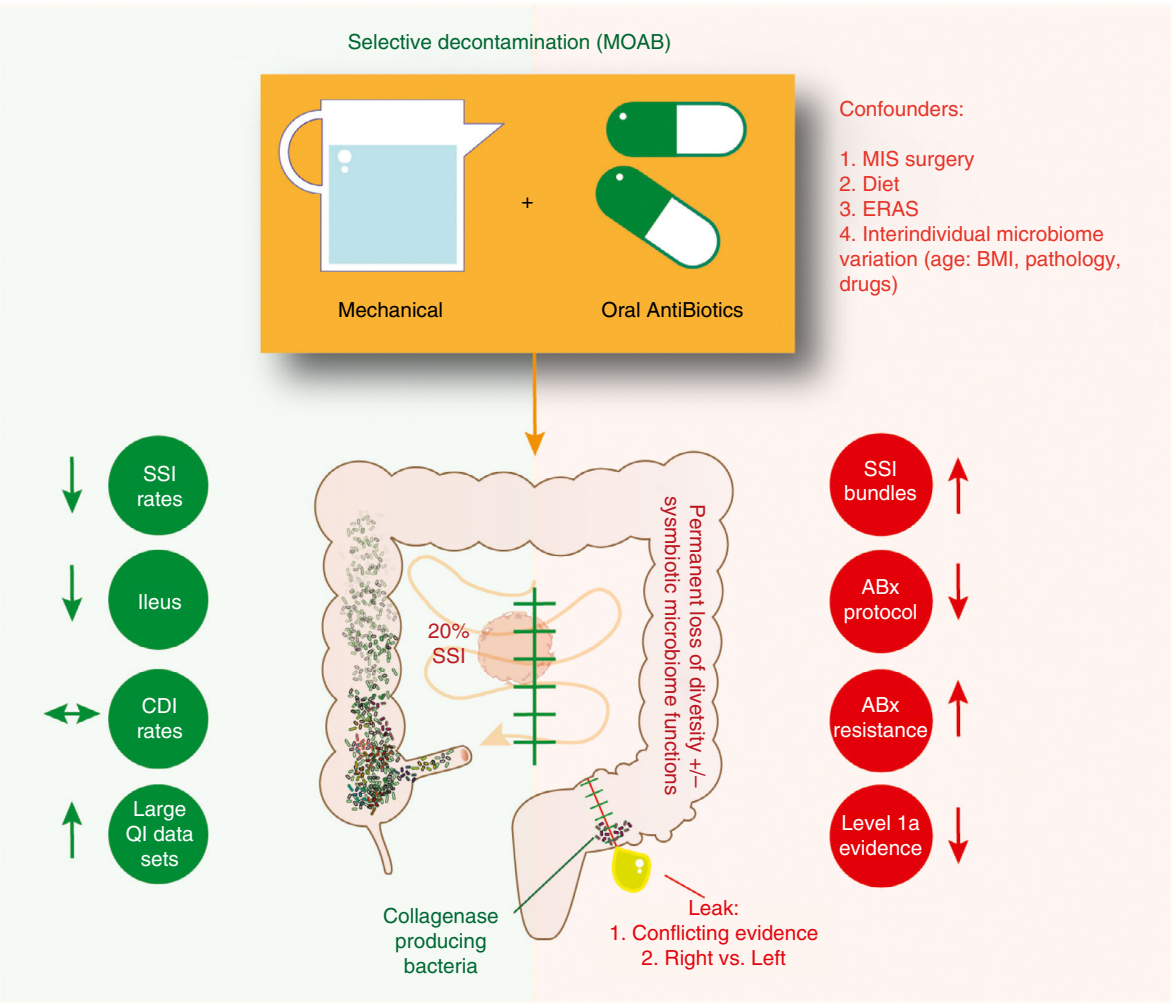
Arguments for and against the use of mechanical bowel preparation and oral antibiotics in elective colorectal resection**.** MOAB, mechanical bowel preparation and oral antibiotics; MIS, minimally invasive surgery; ERAS, enhanced recovery after surgery; BMI, body mass index; SSI, surgical site infection; ABx, antibiotics; CDI, *Clostridium difficile* infection; QI, quality improvement.

A long‐standing area of controversy is the use of mechanical bowel preparation and oral antibiotics (MOAB) prior to elective colorectal resection 11, 12. Marked differences exist between clinicians worldwide 13. Recent guidelines from the American Society of Colon and Rectal Surgeons strongly recommend the use of MOAB in elective colorectal resection to reduce SSI 14, 15. Other international bodies have recognized the increasing body of evidence and altered their recommendations in a more conservative manner but stopped short of endorsing this practice because of the lack of Level 1 evidence 16. This paper summarizes the arguments for and against the use of MOAB in elective colorectal resection, highlighting the areas of controversy and evidence gaps, and provides pragmatic suggestions for colorectal practice (Fig. 1).

## Arguments supporting the use of MOAB

The combination of MOAB in elective colorectal resection is associated with lower rates of SSI. Numerous reports from the North American Surgical Quality Improvement Program (NSQIP) show improved clinical outcomes after varying versions of preoperative MOAB 17, 18, 19, 20, 21, 22, 23, 24, 25, 26, 27, 28, 29, 30, 31. These observational studies include many thousands of patients undergoing elective surgery and show that the combination of MOAB in comparison to MBP alone is associated with a reduction in SSI of about 50%. Similar reductions are seen in numerous randomized controlled trials (RCTs) 32, 33, 34, 35, 36, 37, observational studies 13 and meta‐analyses, both in meta‐analyses of RCTs alone 2, 38, 39, 40 and in those analysing both RCTs and observational studies 41, 42.

There seem to be additional patient benefits in using MOAB. Over the last decade, accumulating reports have highlighted reductions in anastomotic leak (AL), ileus, readmission rates, reoperation rates and even mortality. However, these findings are not consistent across all studies. Positive effects of MOAB have been seen in multiple retrospective studies 13, 18, 19, 21, 22, 23, 24, 25, 26, 27, 28, 29, 30, 31, 43. The beneficial effects may be more evident in left‐sided colonic resections or rectal resections 22, 29, 44. Several underpowered RCTs, however, have shown no difference in AL rates 36, 45. Meta‐analyses vary in their conclusions regarding the additional benefits of MOAB. No differences in AL rates were seen in a meta‐analysis including 16 RCTs published between 1979 and 2007 38 nor in a recent network meta‐analysis including 8458 patients in 38 RCTs, whereas significant multiple additional benefits were reported in two recent large meta‐analyses, although these effects were less evident when oral antibiotics (OAB) were considered alone 41, 42. Developing evidence implicating intraluminal bacteria in the pathogenesis of AL and reduction in AL with locally administered antibiotics and selective gut decontamination regimes 46 may go part way to explaining the reduction in AL seen with MOAB.

The consequences of infectious complications, such as SSI and AL, may persist for years and are associated with a reduction in quality of life years after the initial surgery 47. There is extensive evidence to show that SSI is a risk factor for the development of incisional hernia 48, 49, 50 which is a common, complex and costly complication of colorectal surgery associated with considerable morbidity. Septic complications increase the permanent stoma rate and in rectal resections supposedly temporary diverting ileostomies are not reversed in more than a third of cases 51, almost always due to a septic anastomotic complication. There is also evidence to support increased risk of local and distant cancer recurrence and reduced survival following AL 52, 53, 54, 55, 56.

Reducing these complications by using MOAB is therefore a very attractive proposition yet possible widespread reintroduction of this policy has raised concerns that the incidence of *Clostridium difficile* related infections (CDI) may rise. A large body of evidence refutes this concern. Only two published studies show an increase in CDI rates 57 or readmissions due to CDI 18, whereas two retrospective studies show a beneficial effect. Kim *et al.* 19 showed that patients having MOAB had a lower CDI rate than those with no bowel preparation (0.5% *vs* 1.8%, *P* = 0.01) while Al‐Mazrou *et al*. 58 showed a similar reduction with OAB alone. The majority of studies show no differences in CDI rates in patients exposed to OAB. This holds true for retrospective studies 30, 31, 59, 60, 61, RCTs 33, 34, 35, 36, 37, 45 and in meta‐analysis 42. There is no evidence of harm in terms of increase in CDI rates by using MOAB.

## Arguments against the use of MOAB

There is still a lack of high quality supporting Level 1 evidence, coupled with concerns around antimicrobial stewardship, choice of antibiotics, negative patient experience and the unknown, little understood, short‐, medium‐ and long‐term consequences on the microbiome and what this may mean for other oncological outcomes.

The majority of the evidence showing benefit from MOAB is from big datasets, such as NSQIP and the European Society of Coloproctology (ESCP) snapshot audit 62. The North American registry data have influenced the American guidelines strongly where it is summarized as Level 1b (strong recommendation; moderate quality evidence) 15. This recommendation arises from the inclusion of ‘exceptionally strong evidence from observational studies’. However, these data have been criticized 11 for use of multiple retrospective reports using the same overlapping datasets and heterogeneity between groups. In many reports, the groups that receive MOAB tend to be younger, fitter, with fewer comorbidities, lower corticosteroid use and earlier stage disease which may contribute to their lower SSI rates. Despite the large numbers in these datasets, this evidence may not be robust.

Irrespective of data quality, arguments against the routine introduction of MOAB prior to elective bowel resection centre around the fact that SSI reduction bundles alone can achieve low rates of SSI without necessarily including MOAB. In addition, it is also questioned whether OAB alone are enough to reduce SSI. In a similar vein, any potential reduction in AL rates achieved by implementation of safer anastomosis bundles (ongoing ESCP EAGLE study) or adoption of new technology (ongoing IntAct study) may make possible reductions in AL through use of MOAB less relevant.

Evidence based care bundles focused on reducing SSI rates have been successfully implemented in many institutions over the last 10 years. In the UK, SSI bundles have been created based on guidelines from the National Institute for Health and Care Excellence, the World Health Organization and Health Protection Scotland with additional components added from the published literature 4, 63, 64. In the USA, similar national guidelines have been formulated by the American College of Surgeons and the Centers for Disease Control and Prevention 65, 66. Application of SSI care bundles form a recommended component of enhanced recovery guidelines 14.

The content of SSI care bundles varies but will contain common and variable components. Common shared components include prophylactic intravenous antibiotics, preoperative bathing, hair removal and maintenance of normoglycaemia and normothermia. Less uniformly used interventions include smoking cessation, MRSA screening, 2% alcoholic chlorhexidine skin preparation, wound protectors, antibiotic impregnated sutures, change of gloves and instruments prior to skin closure, novel wound closure devices and MOAB. Compliance to care bundles can be challenging and resource intensive 67 and requires continual audit and real‐time feedback to alter practice 68. Financial incentives and penalties in North America surrounding potentially preventable SSIs have resulted in significant reductions in SSI in major institutions showing that this can be achieved in practice and sustained over time 69, 70.

Implementation of SSI care bundles has been shown to reduce SSI rates by up to 40% 68, 69, 71, 72, 73, 74. The majority of the published studies are cohort studies. However, two RCTs have been carried out 75, 76. Despite clear heterogeneity between these studies two meta‐analyses have also demonstrated the effectiveness of SSI care bundles 5, 77. The results clearly show that SSI bundles are effective in reducing SSI rates irrespective of whether MOAB form part of the bundle. Individual institutions can achieve rates of SSI with implementation of SSI reduction bundles as low as 1.8% 78. However, arguably it is more important to define which of the components of SSI bundles contribute the most to the effectiveness of the intervention. A recent meta‐analysis 77 attempted to address this through sub‐group analysis and identified MOAB, a separate sterile instrument closure tray and glove change prior to closure as providing ‘significantly greater SSI risk reduction’. Interestingly, the one study in this meta‐analysis that failed to show effectiveness of bundle implementation was a well‐designed RCT which omitted MOAB from the experimental arm of the trial. These studies also suffer from differences in compliance levels and how SSI rates were calculated as well as publication bias. Cumulatively, SSI bundles are evidently effective and should be standard of care 79.

It has been assumed that MBP is a requirement in order for OAB to be effective. The administration of MBP prior to elective colectomy is considered unpleasant by many patients, but few studies have taken patient satisfaction into consideration. Taking bowel preparation, often for the second time in a few weeks, may add considerably to the anxiety and distress experienced by the patient before major elective colorectal surgery. The available bowel cleansing agents are often poorly tolerated, time consuming and have unpleasant side‐effect profiles, resulting in reduced compliance and poor bowel preparation 80. Preparations containing polyethylene glycols are diluted in large volumes of water (up to 4 l) and have an unpalatable taste 81. Elderly patients, in particular, find it hard to drink the large volumes of fluid required 82. Sodium phosphate preparations are better tolerated due to the smaller volume of liquid (300 ml water) and palatability 83 but are associated with safety concerns such as major fluid and electrolyte shifts and so should be avoided in patients with chronic kidney disease, congestive cardiac failure, cirrhosis or in patients with electrolyte disturbances 81. Difficulties in administering MBP may also be anticipated in other patient groups including patients with poor reading skills, immobility or frailty or patients taking multiple medications.

Patient concerns about taking MBP underline the need to question whether SSI reduction may be brought about by OAB alone. The need for an RCT to determine this has long been discussed 17, 84, 85, because data on OAB alone are contradictory. Different reports show that the use of OAB alone is worse than 24, 25, 27, 29, 30, 31, 43, 86, equivalent to 22, 26, 28, 42, 87 or better than 17, 20, 32 the MOAB combination in reduction of SSI. The problems are a lack of RCTs that focus on OAB alone in the absence of MBP, small numbers in the OAB alone groups and selection bias in the cohort studies. In the largest and most recent meta‐analysis 42, SSI rates were compared between patients having MOAB and OAB alone. Four studies were included in this analysis, two RCTs (*n* = 709) and two cohort studies (*n* = 22 774), with no difference in the incidence of SSI between these groups overall or when the RCTs and cohort studies were considered separately. So, the use of OAB alone does seem to reduce SSI by at least an equivalent level to MOAB. This is reinforced by a further comparison considering OAB alone *vs* no preparation in two cohort studies including 16 390 patients with SSI reduced in the OAB group (relative risk 0.56, 95% CI 0.38–0.83, *P* = 0.004). In the Netherlands, a cohort study assessing OAB alone as standard of care over time was able to demonstrate a 6.2% reduction in deep SSI and/or mortality, which equated to a 42% risk reduction 88. The authors question the benefit of MBP in addition to OAB and are running the PreCaution study to assess whether use of OAB alone is sufficient 89.

There is little Level 1 evidence for OAB alone, so should we wait to change practice until ongoing RCTs report? The first Level 1 evidence including a no bowel preparation arm is the report of the MOBILE study 45. Patients undergoing colonic, but not rectal, surgery were randomized 1:1 to MOAB or no bowel preparation. Of the 417 patients randomized, there was no difference in the primary end‐point of SSI at 30 days postoperatively (7% *vs* 11%, odds ratio 1.65, 95% CI 0.80–3.40, *P* = 0.17). In the MOBILE study, low SSI rates may have been due to a high proportion of laparoscopic resections, high case exclusion rate, and more than 50% of resections being right‐sided, with the suggestion that the low SSI rates may have meant it was underpowered 90, 91. However, this study does not address the issue of whether OAB alone reduce SSI. This is being examined in several large, well‐powered, ongoing RCTs. The PreCaution 89, COMBINE 92 and SELDDEC trials include both colon and rectal resections; the REaCT‐NSQIP and COLONPREP trials examine colonic resection alone. These large trials will provide further evidence about the role of OAB alone. However, the inclusion of both colon and rectal resections together introduces heterogeneity and possible difficulty with interpretation as OAB alone may be preferred in colonic surgery and MOAB in rectal surgery 93. However, the use of MOAB in rectal surgery will be addressed by the PREPACOL2 study. There is also a concern that these trials will end up being underpowered as they assume SSI rates in the control arms in the region of 15% and aim to show a 40%–50% reduction in primary outcome with the intervention. As SSI rates fall more widely, due to better awareness and control of SSI risk factors, the statistical assumptions on which the power calculations are based will be challenged. The relative benefit on SSI of MOAB or OAB alone, if proven, may only be small within the context of well‐implemented SSI reduction bundles.

The choice of antibiotics to be employed for MOAB or OAB prophylaxis is unclear. Huge numbers of antibiotics and antibiotic combinations have been employed in clinical trials. The Cochrane review of antimicrobial prophylaxis in colorectal surgery identified 68 different antibiotics in the 260 trials included. The use of multiple different drugs, regimes and site of application, including intraluminal 94, make it impossible to conclude if any regime is better or worse than any other. It does, though, appear clear that combination of aerobic and anaerobic cover is important. Concerns about excessive and widespread use of antibiotics leading to antibiotic resistance have led to programmes such as ‘Start Smart and then Focus’ within the UK 95. Despite guidelines, national surveys show that adherence to surgical prophylaxis is poor, both in timing and duration 96.

As well as concerns for antibiotic stewardship, the widespread reintroduction of MOAB or OAB alone in elective colorectal surgery may have unintended consequences on the human microbiome, although this may be offset by a subsequent reduction in the use of broad spectrum antibiotic courses to treat SSI. The human large bowel microbiota comprises a consortium of many hundreds of bacterial species that carry out an array of enzymatic reactions, many distinct but essential to human genome encoded activities. In essence, therefore, humans possess an ‘extended genome’ of hundreds of millions of microbial genes located in the intestine, known as ‘the microbiome’ 97. The microbiome is highly individualized and niche specific, which may in itself explain much of the conflicting data from both MBP and MOAB trials. We have limited mechanistic data to explain how MOAB actually reduce SSI rates, as almost all prospective trials have completely failed to account for the microbiome and its functions. Recent data suggest that the commensal bacterium *Enterococcus faecalis* contributes to the pathogenesis of AL through its capacity to degrade collagen and to activate tissue matrix metalloproteinase 9 in host intestinal tissues 98. The conclusion of this work does not support the wholesale destruction of the gut microbiome, but rather a precision guided approach that knocks down specific strains or their functions at the site of surgical pathology.

Antibiotics have a dramatic and long standing impact on both the structure and function of the gut microbiome that lasts well beyond the surgical intervention 99. By fundamentally altering (perhaps permanently) the gut microbiome with MOAB or OAB in the context of surgery it is possible that we are inadvertently modifying patient response to adjuvant therapy 100 and drug metabolism 101, 102, 103, adversely influencing their risk of non‐communicable disease, drug toxicity, or even irrevocably altering gut function that may have a deleterious impact on quality of life. Currently, none of these end‐points is measured in MOAB trials. While massive destruction of a complex and delicate ecosystem vital for human health and recovery from surgery should not be undertaken without careful consideration as it may have unpredictable consequences that lead to patient harm, it needs to be balanced against the reduced use of broad spectrum therapeutic antibiotics to treat SSI. Moving forwards, choice of bowel preparation must adopt a personalized strategy that promotes the beneficial behaviours of an individual’s commensal organisms and suppresses pathobionts that drive surgical complications 90, 104.

## Conclusion

There is strong evidence that SSI reduction bundles are effective and should be used routinely in the elective colorectal surgery pathway. Colorectal units should monitor SSI rates and implementation of SSI bundles should be audited while aspiring to the low SSI rates known to be achievable from published work.

There is a large and increasing body of evidence showing that MOAB are associated with reduced SSI and other postoperative complications but the quality of this evidence and its subsequent weighting is debated. It is possible that any observed effect on reduced SSI rates may be due simply to the use of OAB alone rather than MOAB. It is conceivable that, in time, OAB may be preferred in colonic surgery and MOAB in rectal resections. Informing patients of the possible benefits and risks and involving them in shared decision‐making to use OAB or MOAB is recommended as best current practice due to the considerable uncertainty that persists.

Emerging research focusing on the microbiome is likely to guide more personalized and specific bowel preparation regimes which will target reduction of both AL and SSI. It is imperative that clinicians contribute to ongoing research and offer their patients the opportunity to participate in high quality research studies designed to fill the existing knowledge gaps.

Whilst there is much that is still unknown, the use of MOAB appears to be safe and could reasonably be used as part of an audited SSI reduction bundle, with the caveat that its use may need to be adjusted as results of ongoing high quality research emerge.

## Conflicts of interest

No conflict of interest: SE Duff, CLF Battersby, RJ Davies, L Hancock, J Pipe, S Buczacki, J Kinross, C Walsh. AG Acheson: in the last 3 years my research department has received grant support from Pharmacosmos, Denmark and Vifor Pharma, Switzerland. Honoraria or travel support received for lecturing from the following companies: Olympus, Essex, UK, Vifor Pharma Ltd, Glattbrugg, Switzerland, and Pharmacosmos, Denmark.
